# Mediation analysis to unravel mechanisms underlying association between platelet transfusion and postoperative delirium

**DOI:** 10.1186/s13054-016-1513-0

**Published:** 2016-10-28

**Authors:** Bingwen Zhang, Zhongheng Zhang

**Affiliations:** 1 Department of Critical Care Medicine, Jinhua Municipal Central Hospital, Jinhua Hospital of Zhejiang University, 351#, Mingyue street, Jinhua, 321000 Zhejiang Province China; 2 Department of Emergency Medicine, Sir Run-Run Shaw Hospital, Zhejiang University School of Medicine, No. 3, East Qinchun Road, Hangzhou, 310016 Zhejiang Province China

We read with great interest the study by Rudiger and colleagues [1]. It is a retrospective study investigating risk factors of postoperative delirium in patients undergoing cardiopulmonary bypass surgery. The authors collected many perioperative potential risk factors and then performed univariate and multivariate analyses to investigate their association with the development of delirium. This is the standard method for risk factor analysis [2]; however, it has long been criticized for a substantial proportion of false positive results. Let us suppose that an investigator chooses 20 factors but none of them has an association with the outcome; regression analyses may obtain positive results a substantial number of times. A method to address this problem is to validate the regression model in an independent cohort (e.g., external validation). However, with the limited sample size and single center nature of the study, this validation cohort was not used.

An alternative approach to such data-driven analysis is hypothesis-driven analysis. Data-driven analysis is performed without the prior hypothesis that it examines the association between risk factors and outcome. Positive associations are reported depending on the results of statistical results. On the other hand, the hypothesis-driven approach is to make a hypothesis before data collection, and data are then used to test whether the hypothesis is supported by the empirical data. An interesting method is the mediation analysis [3]. In the study cited, we could hypothesize that duration of cardiopulmonary bypass is an initiator which causes delirium via the inflammatory pathway, and that inflammatory responses can be measured by platelet indices and C reactive protein [4]. Other pathways also include the tissue hypoxia that can be characterized by lactate and hemodynamics. Once a specific pathway is determined, empirical data can be utilized to test whether the theory is supported by data. Mediation analysis is suitable for the analysis. Alternatively, if the sample size is large enough, the structural equation model can also be helpful to unravel complex relationships between risk factors and outcomes.

Our previous study found that lactate was associated with postoperative occurrence of acute kidney injury [5]. The possible underlying mechanism is tissue hypoxia during and after operation. It is possible that tissue hypoxia may have universal adverse effects on organ functions. Delirium is a type of acute organ dysfunction involving the central nervous system, and thus can be affected by hypoxia. Consistent with this hypothesis, the study showed that lactate levels on intensive care unit (ICU) day 1 were significantly associated with delirium (*p* = 0.008).

## Abbreviation

ICU: Intensive care unit
