# Supplementary material for: Mediation analysis to unravel mechanisms underlying association between platelet transfusion and postoperative delirium
Source: Crit Care. 2016 Oct 28;20:342. doi: 10.1186/s13054-016-1513-0 (PMC5084342; doi:10.1186/s13054-016-1513-0)
Supplement: Related texts

# Reply: Authors’ response

**Authors:** A. Rudiger, M. Schubert, B. Seifert, D. R. Spahn, D. Bettex

We have recently published in *Critical Care* that delirium occurred in 26 % of consecutive ICU patients undergoing cardiac surgery [1]. In this study, patients with and without delirium differed significantly in disease severity scores, intraoperative variables, postoperative laboratory parameters, and the utilization of ICU resources. Focusing on intraoperative variables, cardiopulmonary bypass duration, low mean arterial blood pressure, low hemoglobin levels, low body temperature, high noradrenaline requirements, and transfusion of red blood cells and platelets were significant intraoperative risk factors. In the multivariate analysis, only platelet transfusions remained independent. This suggests that the interplay of different events during cardiac surgery might promote postoperative delirium.

Regarding our findings, Zhang and Zhang highlight in their letter that repetitive statistical testing bears the risk of false positive results [6]. In addition, the authors mention that a hypothesis-driven study has advantages over a data-driven analysis. Mediation analysis is another suggested option.

We thank the authors for these comments and would like to add that a retrospective analysis can only reveal strong associations but does not give answers on causality. Nevertheless, findings from observational studies remain interesting and clinically important for the following reasons: First, observational studies represent the real world whereas randomized controlled trials often exclude important patient groups. As a consequence, well-designed observational studies do not systematically overestimate the magnitude of the associations between exposure and outcome as compared with the results of randomized controlled trials of the same topic [7]. Second, the realization of randomized controlled trials in critically ill patients is challenging, partly due to ethical and administrative constraints, and partly because of the complex interplay between underlying diseases, pathophysiological deteriorations, and therapeutic interventions [8].
